# Design and Implementation of an Atrial Fibrillation Detection Algorithm on the ARM Cortex-M4 Microcontroller

**DOI:** 10.3390/s23177521

**Published:** 2023-08-30

**Authors:** Marek Żyliński, Amir Nassibi, Danilo P. Mandic

**Affiliations:** Department of Electrical and Electronic Engineering, Imperial College London, London SW7 2AZ, UK; a.nassibi15@imperial.ac.uk (A.N.); d.mandic@imperial.ac.uk (D.P.M.)

**Keywords:** edge computing, machine learning, wearable devices, atrial fibrillation detection

## Abstract

At present, a medium-level microcontroller is capable of performing edge computing and can handle the computation of neural network kernel functions. This makes it possible to implement a complete end-to-end solution incorporating signal acquisition, digital signal processing, and machine learning for the classification of cardiac arrhythmias on a small wearable device. In this work, we describe the design and implementation of several classifiers for atrial fibrillation detection on a general-purpose ARM Cortex-M4 microcontroller. We used the CMSIS-DSP library, which supports Naïve Bayes and Support Vector Machine classifiers, with different kernel functions. We also developed Python scripts to automatically transfer the Python model (trained in Scikit-learn) to the C environment. To train and evaluate the models, we used part of the data from the PhysioNet/Computing in Cardiology Challenge 2020 and performed simple classification of atrial fibrillation based on heart-rate irregularity. The performance of the classifiers was tested on a general-purpose ARM Cortex-M4 microcontroller (STM32WB55RG). Our study reveals that among the tested classifiers, the SVM classifier with RBF kernel function achieves the highest accuracy of 96.9%, sensitivity of 98.4%, and specificity of 95.8%. The execution time of this classifier was 720 μs per recording. We also discuss the advantages of moving computing tasks to edge devices, including increased power efficiency of the system, improved patient data privacy and security, and reduced overall system operation costs. In addition, we highlight a problem with false-positive detection and unclear significance of device-detected atrial fibrillation.

## 1. Introduction

Wearable devices have significantly transformed the way we approach health monitoring, as they have allowed individuals to track their health outside of traditional medical settings. Unobtrusive and user-friendly devices have made it possible to predict health events and monitor, among other items, movement disorders, heart rhythm, blood oxygen saturation, cardiovascular function, sleep patterns, neurological disorders, and mental health issues [1,2]. Applications also include fitness tracking, which has become popular for promoting a healthier lifestyle. To this end, processing and analysis of a given continuous stream of real-time physiological data generated by wearables needs to be increasingly performed within wearable devices [3].

Currently, IoT healthcare systems operate on a cloud-based architecture, where data from the devices are sent to a cloud infrastructure for processing and storage. Some of the major limitations with existing systems are security issues, high power consumption, and limited availability of computation power and data transfer bandwidth [4]. Another concern is related to the quality of remotely acquired data, which can be susceptible to corruption by issues related to the hardware, software, device connectivity, and user errors [5]. To ensure accurate and reliable insights within the rapidly growing landscape of complex health care data, data quality assessment and anomaly detection methods are employed [6].

In addition, the exchange of substantial amount of information amongst billions of devices creates a massive energy consumption [7]. One of the energy efficient strategies is edge computing, where data are processed on microcontrollers within the wearable devices. Edge computing also saves time, improves privacy, and reduces network traffic [8].

AF is as the most prevalent arrhythmia and significantly contributes to cardiac morbidity and mortality [9]. It is characterized by irregular atrium activation, with a higher activation rate than in normal sinus rhythm which disturbs normal atrial contraction. Atrial fibrillation, occasional anomaly, is difficult to be detected. For reliable diagnosis long-term high-quality recording is required [10].

The two most common recording techniques for detecting arrhythmias in smart wearable devices, such as smartwatches, are the photoplethysmography (PPG) and electrocardiography ECG [11]. PPG-based AF detection proves to be more intricate compared to ECG-based methods. However, it offers extended monitoring durations and comes at a lower cost compared to ECG-based approaches [12]. In a meta-analysis by Hermans et al. [13], PPG-based devices demonstrated a sensitivity range of 91.5% to 98.5% and specificity ranging from 91.4% to 100%. For ECG-based devices, the reported sensitivity ranged from 94.0% to 98.0%, and specificity spanned from 76.0% to 95%. Wearable technologies represent a significant frontier in health assessment [14].

In this study, we have designed and evaluated the implementation of several classifiers aimed at detecting Atrial fibrillation (AF) on a general-purpose ARM Cortex M4 microcontroller (STM32WB55RG). We compare the performance metrics and computation complexity of different classifiers available in the CMSIS-DSP library (naïve Bayes and SVM classifiers, with different kernel functions).

We have developed a code that simplifies the utilization of this workflow; our script automatically trains chosen classifier and generates a C-header file that can be directly used in a C project. The code is publicly available in the GitHub repository: https://github.com/Marower/Transfer-scikit-learn-models-to-CMSIS-DSP-library (accessed on 25 August 2023). In addition, the scripts to transfer users’ Python model (trained in Scikit-learn) to C environment have been developed and are available in the repository. The microcontroller and Python code for this study can also be find in the repository.

Our aim was to implement these classifiers on our own hearable device, which acquires ECG signals via electrodes positioned inside the ears [15]. To this end, we set out to integrate the classifier with a real-time R-peaks detection algorithm, such as the Deep Matched Filter [16]. This amalgamation aims to enhance the accuracy and reliability of our AF detection system within the context of the hearable device.

The contributions of this work are presented as follows:

- Design and evaluation of machine learning methods for AF detection. These methods include Support Vector Machine classifiers with different kernel functions: linear, polynomial, radial basis function (RBF), and sigmoid, as well as a Naïve Gaussian Bayesian estimator. The evaluation was performed on a general-purpose ARM Cortex-M4 microcontroller.
- Our findings indicate that the SVM with RBF kernel function offers the optimal combination of accuracy, memory consumption, and computation time among the tested methods.
- An open-source pipeline for deploying machine learning methods (SVM and Naive Bayes) on ARM microcontrollers using the CMSIS-DSP library.

## 2. Related Works

Randazzo et al. [10] developed a wearable device specifically designed for ECG acquisition and Atrial Fibrillation (AF) detection. Their approach employed of a simple embedded algorithm whereby R-peaks were initially extracted employing the Pan-Tompkins algorithm. Subsequently, if rhythm fluctuations surpassed predetermined thresholds over time, the recording was categorized as indicative of AF.

Atrial Fibrillation (AF) detection can be achieved by assessing the variability of R-R intervals through machine learning classifiers. Patel et al. [17], for instance, employed a set of 21 features derived from RR intervals. They evaluated 11 distinct machine learning classifiers including Logistic Regression, Linear Discriminant Analysis, Quadratic Discriminant Analysis, K-Nearest Neighbors, and decision tree classifiers trained with various algorithms. Notably, their findings highlighted that the highest accuracy was attained by the model trained using the Extreme Gradient Boosting method, reaching an accuracy of 96.3%.

Neural network models offer a robust means of accurately identifying Atrial Fibrillation (AF) in ECGs captured by portable devices. Marinucci et al. [18] conducted an assessment of a neural model constructed using fully connected dense layers and incorporating 19 features. These features encompassed 4 derived from RR variability, 11 from mean heartbeat ECG morphology, and 4 based on the F wave. Their evaluation was centered around the area under the curve (AUC), revealing that the AUC for the testing dataset stood at 90.8% (with a confidence interval of 88.1–93.5%).

Deep neural networks have found utility in classifying arrhythmias within embedded wearable devices [19]. Nevertheless, a key challenge with deep neural network models lies in their memory footprint, which often surpasses the capabilities of compact wearable devices. To address this, Lee et al. [20] employed ResNet and MobileNet models. To make them compatible with embedded devices, they executed model compression through TensorFlow Lite. This process reduced the model size from an initial 743 MB to a mere 76 KB. Notably, their reported accuracies were impressive, with 98.2% for ResNet and 97.9% for MobileNet when applied to ECG signals sampled at a frequency of 100 Hz.

## 3. Edge Computing

At present, a medium-level microcontroller based on the ARM Cortex-M4 architecture can perform advanced digital signal processing and edge computing. The ARM Cortex-M4 is a low-cost, high-performance embedded processor with floating-point unit (FPU) and digital signal processing (DSP) blocks [21,22]. The scalability and power efficiency of a microcontroller built on the Cortex-M4 architecture make it an excellent choice for wearable applications.

ARM developed the CMSIS DSP Software Library that provides several digital signal processing functions such as signal filtering with finite impulse response and infinite impulse response filters, matrix operations, and statistical and even quaternion functions [23]. The Cortex-M4 is used in several microcontrollers and systems-on-chip (SoCs) with Bluetooth Low Energy technology, such as STM32WB (STMicroelectronics) and NRF52 (Nordic Semiconductors). These microcontrollers and SoCs are formidable options for robust wearable systems. Moreover, microcontrollers with ARM Cortex-M4 cores have the capability to efficiently execute neural network kernel functions [24]. At present, it is becoming viable to integrate low-cost general-purpose microcontrollers and SoCs within a small form factor, such as a smart watch, to achieve a complete end-to-end solution: from signal acquisition through digital signal processing to a detector for cardiac arrhythmias [25].

Mainstream microcontrollers are capable enough to perform machine learning classification [26] or even run deep neural network models; for example a Convolutional Neural Network (CNN) model trained with TensorFlow can be deployed on the Cortex-M4 microcontroller. Sailesh et al. [27] provided a framework that automates code generation for a CNN model on a microcontroller. However, the limited memory on IoT devices restricts the utilization of CNNs in the IoT. Deep neural network models usually require megabytes, while microcontrollers provide kilobytes of memory [28]. For instance, the STM32WB55RG microcontroller, which was used in our study, has up to 256 KB of SRAM. Although it is possible to extend the memory using additional integrated circuits, this would result in increased device size and power consumption.

There are several viable strategies for optimization of deep learning models to run on edge devices [29]:

- Factorization—adding a new layer and splitting the original weight matrix into two lower-rank weight matrices. This results in reduced memory usage but increases computation cost.
- Pruning—removing small-weight connections (below an arbitrary threshold). This method is supported by TensorFlow, but it increases training time and results in a sparse matrix.
- Quantization—reducing the size of the weights. For example, the AlexNet reduction in the size of weights in CNN layers from standard 32-bits to 8-bits and to 5-bits in dense layers did not change the accuracy of the model [30].

In addition, different optimization methods can be combined. These strategies aim to reduce memory consumption but at the cost of code complexity and increased computation time. The optimization of the deep neural network architecture is crucial, as increasing the depth of the network significantly increases the number of operations, computation time, number of network parameters, and demand for microcontroller memory [31].

## 4. Method

We trained five classifiers using Python and Scikit-learn, including four different Support Vector Machine (SVM) classifiers with different kernel functions (linear, polynomial, RBF, and sigmoid) and a Naïve Gaussian Bayesian estimator. The SVM classification is based on a hyperplane, built using the kernel function, that maximizing the margin between classes [32]. The Naïve Bayesian method classifies an observation based on a probability calculated using Bayes’ theorem [33]. The parameters of trained models were saved as C header files and utilized to initialize CMSIS-DCP classifiers.

To train and evaluate the models, we used part of the data from the PhysioNet/Computing in Cardiology Challenge 2020 [34]. We used the data set provided in the first step of the challenge, which contains recordings of a 12-ECG lasting from 6 s to 60 s. Recordings are from 6877 subjects (male: 3699; female: 3178) with a sampling frequency of 500 Hz. Recordings are annotated as: normal, AF, first-degree atrioventricular block, left bundle branch block, right bundle branch block, premature atrial contraction, premature ventricular contraction, and ST-segment depression or ST-segment elevated. One recording can have multiple labels. In this paper, we only used recordings annotated as: AF or normal rhythm (2139 subjects).

We conducted a classification of AF based on heart-rate irregularity. In each recording, we extracted seven features based on RR intervals (time elapsed between two successive R-point detections) [35,36]:

- Min, max, mean, median, and standard deviation of RR intervals in recordings;
- The rMSSD—root mean square of successive difference, given by:
  (1) $$rMSSD=\;\sqrt{\sum_{i=1}^{N-1}{\left.{\left(RR\right.}_{i}-{RR}_{i+1}\right)}^{2}}$$
- The pNN50—number of successive differences in RR intervals exceeding 50 ms in a recording.

Our data set was split into a training set (first 1500 subjects) and a test set (last 639 subjects). The training set was used to train the classifiers, and the test set was used to validate the performance of the classifiers on the microcontroller.

The performance metrics of the classifiers were tested on the STM32 Nucleo-64 development board with a STM32WB55RG microcontroller. The test part of the data set was stored in the microcontroller memory. During validation, each classifier was employed to classify every sample, and the results were transmitted for validation against true labels.

The execution time of 10 iterations of the AF classifier on the test data set (6390 recordings) on the STM32WB55RB was measured. We used a built-in 32-bit microcontroller timer with a 1MHz clock source to measure the execution time.

**Figure 1:**
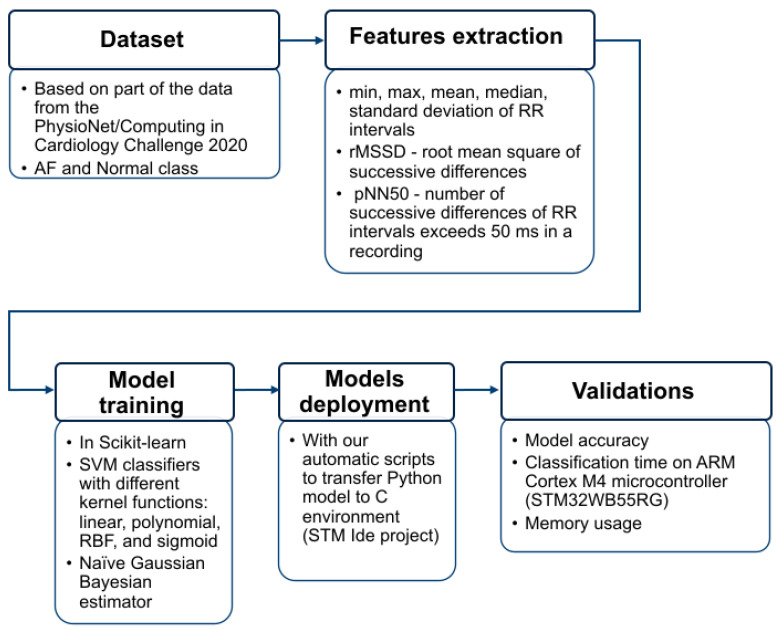
Workflow diagram depicting the estimation of a set of features from a portion of the data from the PhysioNet/Computing in Cardiology Challenge 2020. The selected machine learning models were trained in the Scikit-Learn Python environment, and using our automatic scripts, we deployed the models into the STM project. We validated the models for accuracy, execution time, and memory usage on the ARM Cortex-M4 microcontroller.

The used methodology is summarized in the workflow diagram shown in Figure 1.

We performed a comparison of classification execution times on different systems. We performed the described measurement of classifier execution times on a tablet, a desktop PC, and Imperial College High Performance Computing (HPC) clusters (https://www.imperial.ac.uk/computational-methods/hpc/) (accessed on 25 August 2023). The same Python code was executed on every system. We used an 11-inch iPad Pro tablet with an M1 chip and ran the code as a local Jupyter notebook in the Carnets Plus application (https://apps.apple.com/in/app/carnets-jupyter/id1450994949) (accessed on 25 August 2023). For the desktop PC, we utilized an Intel Core i7-4790 CPU and the code was executed in the PyCharm Community Edition 2022.1.3 application. To run the code on the HPC, we used the Imperial College JupyterHub, and the code was executed in a single-core job with 8 GB RAM.

## 5. Results

**Figure 2:**
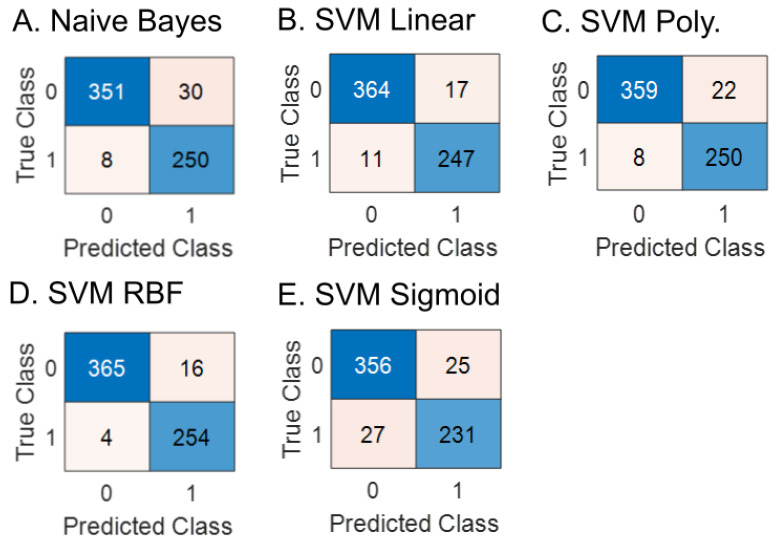
Confusion matrices for the considered classifiers: (**A**) Naïve Bayes classifier, (**B**) SVM Linear classifier, (**C**) SVM Polynomial classifier, (**D**) SVM RBF classifier, and (**E**) SVM Sigmoid classifier.

The confusion matrices for each of the classifiers on the test data set are shown in Figure 2. Among the classifiers, the SVM classifier with a sigmoid function exhibited the lowest accuracy (92.0%). The Naïve Bayes classifier achieved an accuracy of 94.1%. The highest accuracy was observed with the SVM classifier using the RBF function (96.9%). It should be mentioned that the SVM RBF classifier produced 4 false negative and 16 false positive classifications.

The execution times required to complete the classification of the test data set (10 × 639 recordings) by each classifier on the STM32WB55RB are summarized in Table 1. The Naïve Bayes classifier showed the fastest performance with an execution time of 0.417 s, with each recording classified within 65 microseconds. Conversely, the SVM classifier with the sigmoid function proved to be the slowest, requiring 11.966 s. The SVM classifier employing the RBF function exhibited the fastest execution time among the SVM classifiers, completing classification in 4.602 s.

The results of the classification execution time per one recording are summarized in Table 2. The execution time comparison is conducted between a compiled native (C) implementation on the Cortex-M4 and a partly interpreted implementation (Python 3.9 64-bit) on the other devices. The classification performed on the tablet was between 113 to 527 times faster (respectively, for Naïve Bayes and SVM Sigmoid classifier) compared to the ARM Cortex-M4.

Table 3 summarizes memory usage of different classifiers. The Naïve Bayes classifier required only 31 floating-type parameters, while the SVM RBF classifier employed 642 parameters. Notably, the SVM sigmoid classifier exhibited the highest parameter count, reaching 1827. Memory usage is strongly correlated with the computation time of the classifiers but not with their accuracy. Among the tested SVM classifiers, the SVM RBF classifier exhibited the highest accuracy, lowest memory usage, and shortest computation time. Conversely, the Naïve Bayes classifier exhibited the fastest performance, required the least memory, and achieved a moderate level of accuracy compared to the other classifiers assessed.

## 6. Discussion

In our study, the SVM classifier with RBF kernel function exhibited the highest accuracy, with a sensitivity of 98.4% and specificity of 95.8%. These findings are aligned with those previously mentioned in the literature (Table 4). However, it should be noted that direct comparison of results should be avoided due to the fact that classifiers were tested on different data sets. It is also important to note that Tuboly et al. [37] and Tateno and Glass [38] used publicly available data sets, while Tison et al. [39] performed a clinical study to validate a commercially available smartwatch in AF prediction. In their three papers [37,38,39], classification was based on RR intervals, Petmezas et al. [40] conducted classification based on the raw ECG signal, employing a CNN-LSTM Network for the classification process.

Our implementation of machine learning models, without any optimization, required only a few kilobytes (KB) of memory (Table 3); the largest SVM sigmoid classifier utilized only 7.14 KB. Hence, simple machine learning models are a viable option for deployment on edge devices.For the purpose of AF detection, deep neural network models constructed using ResNet and MobileNet architectures demonstrated a remarkably compact size of 76 KB [20].

We demonstrated that the tested classifiers exhibited low computation time based on extracted features, with an average of 65 μs for the Naïve Bayes classifier, 720 μs for SVM RBF, and 1873 μs for SVM with a sigmoid kernel function, for a single recording. This favorable computation time makes the application of the AF classifier on an edge device viable for real-life scenarios. It is worth noting that the R-peak detection in ECG signals is not a computationally demanding task [41]. Nonetheless, transitioning the classification task to more potent devices can lead to a marginal reduction in computation time, as demonstrated in Table 2. For instance, an 11-inch iPad Pro equipped with the M1 chip completes classification utilizing SVM with RBF kernel approximately 253 times faster compared to the ARM Cortex M4 chip.

It is a given that executing tasks as quickly as possible is an energy-efficient strategy [42]. In battery-powered devices, minimizing power consumption is of paramount importance. Swift task execution allows the microcontroller to spend more time in a low-energy sleep state, leading to a reduction in overall energy consumption.

Ensuring fast performance necessitates careful optimization. Different implementations of classifier algorithms exhibited varying performances on edge devices. Profentzas et al. [28] discovered that the CMSIS framework generates the fastest and most energy-efficient code, although it is also the most challenging to employ. In this present study, our Python scripts bridge this gap by providing a user-friendly solution; they generate the C code that is compatible with the CMSIS framework, which can be seamlessly integrated into embedded projects.

The advancement in technology and the increasing data traffic from a growing number of devices have changed the way data are processed. At present, computing is transitioning from centralized servers to edge devices [43]. Performing computing or classification tasks, such as detection of AF, on an edge wearable device instead of transferring all data to the cloud offers several advantages. It is power efficient, reduces costs associated with data transfer and server maintenance, and enhances the security and privacy of users [44]. In our case, a single recording comprises 48,000 bits (6 s × 16 bits per sample × 500 samples per second). Transferring such a substantial amount of data via Bluetooth Low Energy would require at least 24 ms (assuming a maximum bandwidth of 2 Mbit/s), which is longer than performing edge classification with any classifier described in this paper.

On the other hand, moving the classification task from a microcontroller to a tablet, PC, or the cloud greatly reduces computation time (Table 2) when computation times on more-advanced systems are comparable. We found that the tablet was the fastest system in our comparison. The advantage of the tablet over a desktop PC reflects progress in processor design; the Intel Core i7-4790 was introduced to the market in 2014, and the iPad Pro 11-inch with an M1 chip was introduced in 2021. Our simple, small classification task does not utilize the HPC’s advantages, as an HPC cluster is intended to solve large, complex problems (exceeding the computation power of a single computer) or many small problems.

On every system, the Naïve Bayes classifier was the fastest. However, we observed some differences in the performance of SVM classifiers; on the microcontroller and the HPC cluster, the RBF kernel was faster, while on the tablet and the PC, it was the polynomial one. An explanation of this phenomenon exceeds the scope of this paper.

## 7. Atrial Fibrillation Model Improvements

Heart arrhythmia classifiers, such as the AF classifiers described in this work, can be utilized to enhance the efficiency of health monitoring systems [45]. The majority of ambulatory recordings are normal and do not exhibit any arrhythmia; hence, they do not require additional processing and can be dealt with immediately after acquisition. This approach optimizes the use of resources and saves physicians’ time.

On the other hand, the clinical significance of device-detected AF is unclear, as false positives may be potentially harmful to patients [46]. Note that AF is relatively uncommon among individuals under the age of 50; it has been found in 0.5% of people aged 50–59, and its occurrence increases with age. More specifically, it was found in 8.8% of people between the ages of 80 and 89 [47]. In our case, with an assumed representative population of 1000 people 50–59 years old and with 5 AF patients, to match population statistic described in Lip et al. [47], the SVM RBF classifier probably will detect all 5 AF cases but will also show 42 false positives. This will require further investigation. The study on AF assessment performed by Apple showed that AF was present in 34% of the detected irregular pulses that were identified by optical sensors on wearable devices [48]. In their study, out of the total of 419,297 participants recruited, 0.52% of the participants received notifications indicating an irregular pulse.

The effectiveness of screening for AF in the elderly to improve treatment outcomes for screen-detected asymptomatic patients remains unclear [49]. It is uncertain whether such screening provides superior results compared to treatment initiated after detection through usual care or when symptoms develop. Currently, no expert consensus recommends screening for AF [50].

One way of improving AF classifiers involves detecting and analyzing the P-wave in the ECG signal. During an AF episode, atria do not repolarize properly, causing the normal P-wave to be displaced by the fibrillatory F-wave. The P-wave analysis substantially reduces false positives with a minimal decrease in sensitivity of AF classifiers [51]. However, implementing this technique into wearable devices with optical or one-lead ECGs is challenging, as the P-wave is rarely visible in a single lead I configuration, as commonly used in wearable devices such as torso bands or the Apple Watch ECG. However, more-complex devices such as the KardiaMobile 6L (AliveCor) implement a six-lead ECG and result in properly incorporated P-wave information in AF detection.

Another way to improve the accuracy of the AF classifier is to use a complex deep neural network solution. Petmezas et al. [40] used a CNN for automatic extraction of features from ECGs and a Long Short-Term Memory (LSTM) model for accurate classification of ECG rhythm types. They reported that the network for AF classification achieved a sensitivity of 97.87% and specificity of 99.29% using a ten-fold cross-validation strategy. Their proposed model was trained on the MIT-BIH Atrial Fibrillation Database.

## 8. Conclusions and Future Work

We have evaluated five classifiers for the detection of atrial fibrillation based on heart rhythm irregularity. This has been achieved by developing Python scripts to automatically transfer Python models trained in Scikit-learn to the C environment for deployment on microcontrollers; in our case, the performance of these models has been assessed on a STM32WB55 microcontroller (ARM Cortex-M4). We have found that the best performance was achieved by the SVM classifier with the RBF kernel function, with a sensitivity of 98.4% and specificity of 95.8%. In addition, it exhibited efficient computation time, processing each recording in just 720 μs, and required a minimal storage space of 2.5 kB for its parameters. These results have emphasized the feasibility of deploying machine learning classifiers in edge computing environments.

Future work will include:

1. Investigating the deployment of more-advanced AF classifiers on edge devices, including dense and convolutional neural networks. Utilizing these models is expected to enhance specificity and sensitivity [40].
2. Integration of the classifier with online ECG signal analysis by implementing an R-peak detection algorithm on the microcontroller. Of particular interest is a hearable device, such as implementation of R-peak detection in an Ear–ECG [15] with a deep matched filter [16].

## Figures and Tables

**Table 1:** The execution times of 6390 recordings (10 times the test data set) on STM32WB55RB for AF classifiers.

Classifier	Execution Time (s)	Classification Time of One Recording (μs)
Naïve Bayes	0.417	65
SVM Linear	5.684	890
SVM Polynomial	5.578	873
SVM RBF	4.603	720
SVM Sigmoid	11.966	1873

**Table 2:** Execution times for the classification of one recording (μs) on different systems.

Classifier	ARM Cortex-M4 (STM32WB55RG) @ 64 MHz	Tablet iPad Pro 11 in M1 Chip @ 3.2 GHz	Desktop PC Intel Core i7-4790 @ 3.6 GHz	Imperial College HPC Cluster
Naïve Bayes	65.26	0.58	1.09	0.92
SVM Linear	889.51	2.11	3.91	6.15
SVM Polynomial	872.93	1.87	3.28	5.31
SVM RBF	720.34	2.85	7.66	4.98
SVM Sigmoid	1872.61	3.55	9.85	13.29

**Table 3:** Memory usage of different classifiers.

Classifier	Number of Floating Parameters	Memory Size (Bytes)
Naïve Bayes	31	124
SVM Linear	1569	6276
SVM Polynomial	1212	4848
SVM RBF	642	2568
SVM Sigmoid	1827	7308

**Table 4:** The sensitivity and specificity of the SVM RBF classifiers compared with those reported in the literature.

Classifier	Sensitivity $\mathrm{Se}\;=\;\frac{\mathrm{TP}}{\mathrm{TP}+\mathrm{FN}}$	Specificity $\mathrm{Sp}\;=\;\frac{\mathrm{TN}}{\mathrm{TN}+\mathrm{FP}}$
SVM RBF (described in this paper)	98.4%	95.8%
Tuboly et al. [37]	97.6%	93.0%
Tison et al. [39]	98.0%	90.2%
Tateno and Glass [38]	93.2%	96.7%
Petmezas et al. [40]	97.9%	99.3%

## Data Availability

We used part of the publicly available data set from the PhysioNet/Computing in Cardiology Challenge 2020 [34].

## Abbreviations

The following abbreviations are used in this manuscript:

AF	atrial fibrillation
SVM	support vector machine
RBF	radial basis function
CNN	convolutional neural network
IoT	Internet of Things
FPU	floating-point unit
SoC	system on chip
HPC	high-performance computing
ECG	electrocardiogram
PPG	photoplethysmogram
